# Instrumentalized migration: avoiding the trap

**DOI:** 10.12688/openreseurope.18635.1

**Published:** 2024-11-22

**Authors:** Maxime Lebrun, Tanja Ellingsen, Hanne Dumur-Laanila, Sophie Bujold, Annabel Miller, Beth James, Gordan Akrap, Josip Mandić

**Affiliations:** 1 The European Centre of Excellence for Countering Hybrid Threats, Helsinki, Finland, 00130, Finland; 2 Faculty of Social Sciences, Nord University, Bodø, Nordland, 8049, Norway; 3 Ridgeway Information EU, Hague, 2595, The Netherlands; 4 Dr Franjo Tuđman Defense and Security University, Zagreb, 10000, Croatia; 5 University North, Koprivnica, 48 000, Croatia

**Keywords:** Instrumentalized migration, hybrid threats, EU, migration and asylum pact

## Abstract

The article considers the European Union’s (EU) and its Member States’ capacities to face the challenge posed by instrumentalized migration as a hybrid threat activity. Instrumentalized migration in this context entails people being forcibly displaced towards an EU border and made to cross it to claim international protection with an aim of causing capacity overload, adverse reactions, or exerting larger pressure on the target state. Because global migration is a highly politicised and securitized issue in European and domestic politics, authoritarian states may see a strategic opportunity in instrumentalizing it their advantage. Responding strategically to instrumentalized migration requires identifying policy pitfalls and value traps while managing to maintain as many tools and as much space for manoeuvre as possible. Authoritarian states may use instrumentalized migration to further their wider agenda of turning international law into a system of rules which would primarily protect state sovereignty and non-interference at the expense of the international protection of human rights. Responses to instrumentalized migration have impacts and establish precedence in terms of acceptable state practice. Considering this, this article discusses the EU’s new Pact on Migration and Asylum and examines how it can be used to the advantage of Member States when dealing with instances of instrumentalized migration.

## Introduction

The European Union’s (EU) new Pact on Migration and Asylum marks a significant step for EU Member States to streamline their migration and asylum policies. The recent increases in cases of instrumentalized migration make it even more important to improve and streamline processes at the EU and Member States levels. Instrumentalized migration refers to a situation in which state and non-state actors use migrants ^1^ as a tool, either covertly or in plain sight, to exert strategic or political pressure upon a target state. This is employed to create and aggravate existing difficulties in organizations, exacerbate social relations, and polarize political debates. The purpose of this article is to understand the challenge that instrumentalized migration poses as a hybrid threat activity. The article focuses on types of instrumentalized migration as exemplified by the Belarus case in 2021 and the Russia case in 2023. These two cases demonstrate a high level of engineering by the initiating states: people claiming asylum via Belarus and Russia were clearly being used as an instrumentalized migration flow. The two cases are also related in their dimensions of luring and violent coercion. Migrants were misled by false advertising, and they were ultimately subjected to violent treatment, arbitrary detention and confiscation of goods and currency. This string of practices was likely meant as deterrence against turning back, effectively forcing them to attempt crossing the border.

This kind of hybrid threat activity leverages existing political and societal grievances to tilt the balance of international law towards the protection of state sovereignty in a manner that may be at the expense of the standards of international human rights law. Authoritarian states may instrumentalize migration to exploit societal fault lines and steer the target state’s practices in a direction favourable to their own agendas of putting pressure on democracy, liberal values, and the system of international human rights law. Furthermore, states targeted by instrumentalized migration may face significant international pressure as they contend with the need to secure their borders and meet growing reception and processing demands whilst continuing to uphold international commitments that protect the rights and safety of people on the move.

Successfully countering hybrid threat activity requires considering the end goal of the adversary. The short-term consequences of instrumentalized migration may be severe, but the end goal of the adversary is to interlink those implications with longer term consequences. This article contends that policy responses to instrumentalized migration tend to be short sighted and made with the intention of balancing state commitments to international protection with their duty to ensure national security and state sovereignty. We propose to transcend this distinction. Respecting international protection commitments as laid out in the Qualification Regulation is the strategic imperative to adequately responding to instrumentalized migration. It is for this reason that the article proposes that this kind of hybrid threat activity leverages domestic politics in order to alter the nature of international law. Authoritarian states are driven by regime survival and implement policies to this end, which is why the challenge of instrumentalized migration directly relates to international law. Authoritarian states have a strategic interest in promoting a view that international law should favour principles such as non-interference and state sovereignty at the expense of the international protection of human rights. Transcending the opposition between international protection commitments and national security makes it necessary for EU Member States to have as much contingency planning and crisis management in place while simultaneously respecting their commitments to international protection. It is when states are faced with no other option but to default away from international protection that authoritarian states will achieve their fundamental goals. Targeted states should maintain a toolbox that maximises their margin for manoeuvre, but they should be able to scale their responses to an appropriate level depending on the situation. Denying the disruptive effects of instrumentalized migration requires preparing for absorbing the flow of instrumentalized migrants as efficiently and humanely as possible.

The new EU Pact on Migration and Asylum, which entered into force in June 2024, notably contains a Crisis and *Force Majeure* Regulation which lays out a series of options for states targeted by instances of instrumentalized migration to trigger solidarity measures through a process driven by the European Commission (EC). This is a legal innovation, and this article discusses its relevance and fitness for purpose. The EU acting on a more collective level against instrumentalized migration has the potential to make it more resilient and to deter authoritarian states from triggering instrumentalized migration in the first place. Pooling resources together and not letting Member States face only detrimental options has the potential to enable the EU to transcend the opposition between international protection and national security.

While it contextualises the challenge of instrumentalized migration with a number of different examples, the article focuses in greater details on a twofold, fundamental goal of instrumentalized migration as witnessed in Belarus and Russia: undermining the credibility of the model of democratic governance, while tilting state practice so that international law would lean towards safeguarding state sovereignty at the expense of the protection of human rights. In addition to the two aforementioned cases, the article also briefly mentions the case of Morocco and Spain. It considers state practice in countering this challenge, discusses the implication of it, and explores the tension it creates in the principles of international law which authoritarian states seek to leverage to advance their agendas. The second part of the article deals with the tools effectively available at present and in the future to EU Member States to assess the margin of manoeuvre they might utilize. The new EU Pact on Migration and Asylum will be considered under this angle.

## Framing the challenge: forcibly displaced people as instruments of power

According to Teitelbaum (1984) and Weiner (1985), people are being used as instruments of power by states in pursuit of certain goals and as a means of foreign policy. More recently, Greenhill (2010) has characterized the phenomena as “weaponised migration” or “coercive engineered migration”. “Coercive engineered migration” refers to cross-border population movements which are deliberately created or manipulated by other state(s) to induce political, military and/or economic concessions from a target state or states. The leverage created by aggressors may manifest as direct threats or public demands that could overwhelm state capacities, or, more indirect coercion, such as private political blackmail. Petty (2022) argues that “weaponized migration” means that “migration, and particularly mass migration” is used deliberately by states or non-state actors as a “ weapon” of sorts to reach political, economic, military, or any other types of goals. Both Greenhill and Petty’s characterizations of the weaponization of migration rely on the ability of migration to bring about political, economic, and military change; the ability for migration flows to bring about such change is exemplified, for example, in the securitizing effect that the so-called refugee crisis of 2015 has had on European societies.

Instrumentalized migration may come with very different degrees and methods of coercion by the initiator state. A clear example of instrumentalized migration was seen when Morocco enabled the crossing of 8,000 migrants, largely sub-Saharans, into the Spanish enclave of Ceuta. The events came amid diplomatic tensions over the disputed territory of Western Sahara, namely, after Spain admitted the leader of the Polisario Front into a Spanish hospital, which prompted Morocco to state, according to Reuters, that it was a “premeditated act” that would have “ repercussions”. Spain responded by deploying border patrols which saw some people voluntarily return to Morocco, and the events did not result in an increase in arrivals in the long-term.

In late 2021, Belarus brought thousands of migrants, largely Kurds from Syria and Iraq, into the country on the promise of access into the EU. This was a step further compared to previous occurrences targeting Europe as clear evidence showed a deliberate pull to get third-country nationals into the country and channel them towards EU borders. Many migrants were left at the western borders of the EU for months during the winter, while Latvian, Lithuanian, and Polish authorities-imposed pushback strategies. Greenhill causally links Belarus’ actions with the EU’s refusal to recognise Aleksandr Lukashenko as the President following irregular elections, which also resulted in EU sanctions against Belarus. Further, in late 2023, Russia was accused of instrumentalizing migration to exert pressure on Finland amid the rising tensions between Russia and NATO. While Belarus has been accused of working to charter flights from the Middle East to its territory through disreputable travel agencies and fabricated visa prospects to facilitate the movement of migrants to the EU border, Russia’s practices have been more coercive. Migrants were prohibited from ever returning to Russia at the cost of threats on the wellbeing of their families and of forced conscription or other disproportionate measures in retaliation to any future irregular stay on Russian territory. This led Finland to close its entire land border with Russia in November 2023. In July 2024, the Finnish Parliament adopted a new law, allowing for a special legal regime whereby border guards would be enabled to assess asylum seekers’ grounds already at the border. The law is very similar to pushback legislation adopted by Lithuania, for instance. Because they contain the least organic migration elements, the Belarus and Russia examples are raised in this article as prototypes of instrumentalized migration. Both examples are used for the analysis in this article.

Responses to instrumentalized migration create precedence in state practice which has a considerable impact on states’ track records in complying with and interpreting various dispositions of international law. This article proposes that the reason authoritarian states decide to instrumentalize migrants against liberal democratic states is that they consider them to be vulnerable to it. Liberal democratic states are committed to respecting and upholding international law and especially, their obligations related to the international protection of human rights. ( Baylis *et al.*, 2023) Authoritarian states may also consider societies in Europe to be particularly prone to cultural and ethnic divisions and polarization. Political issues around migration and integration have proven debilitating for domestic politics in many European countries, provoking demonstrations and stoking racial hatred and discrimination. Anti-migration rhetoric is also a powerful political mobilization factor for parties whose foreign policy platforms often conform with Russia’s. Accordingly, and as pointed out by Petty (2022), in a significant number of cases, a key objective may simply be to create a crisis that forces targeted countries to take measures contrary to their commitments regarding the international protection of human rights, and on a more general level, international law. An effective and genuine access to the international protection procedure must be ensured in accordance with Article 18 of the Charter of Fundamental Rights of the European Union and the Geneva Convention. Human rights and international protection thus limit the state sovereign border control prerogatives.

The challenge of instrumentalized migration links domestic politics and international law. Authoritarian states can leverage societal fault lines and steer the target state’s practice in a direction favourable to their agenda. Ginsburg (2020) notes that authoritarian regimes share a common goal in international law which is to promote noninterference in the internal affairs of states. This is key to their regime survival and solidity. Authoritarian states promote an interpretation of international law in which states would be as minimally responsible as possible for the way they deal with what they consider to be their internal affairs, such as human rights and individual liberties. In this sense, the protection of state sovereignty at the expense of individual rights and liberties is at the core of the authoritarian agenda. Democratic states, on the other hand, defend human and individual rights as the bedrock of the system of international law designed after the Second World War. Authoritarian regimes seek the primacy of state sovereignty and inviolability at the expense of the international protection of human and individual rights globally. It is when states resort to refoulement or compromise with the requirements of international commitments that authoritarian states can claim a successful utilization of migration as a tool to pressure democratic states. Restrictions to freedom of movement worldwide directly undermines the international protection of human rights, since it establishes precedence that authoritarian regimes can use in justifying their own actions and in decreasing democratic states’ standing in the international arena.

Instrumentalized migration further stresses a tension between two principles of international law. The first principle, *Ex Injuria Non Oritur* means that a legal right cannot arise from wrong. It implies that international law cannot recognize a matter of fact that is the result of a wrongful and illegitimate action. This principle underlies the EU’s nonrecognition of the illegal and illegitimate annexation of Crimea by the Russian Federation in 2014. The second principle, *Ex Factis Jus Oritur,* means that the law is born out of effective situations. This implies that international law generally validates an established situation, an effective matter of fact. The manifest existence of a state within established borders and with a population inside of them is a fact that international law recognizes ( Gignac, 2017). The twin principles that legal rights cannot arise from wrongdoing and that the law is borne out of practice balance each other. The first principle is a safeguard against injustice, while the second protects against disorder. While they cannot arise from wrongdoing, legal principles are shaped or determined based on existing factual circumstances or situations. The tension between those principles of international law appears in calls from states to modify international legal conventions on asylum and migration in light of a “new” situation of instrumentalized migration, which they argue did not exist when the relevant instruments were adopted. The EU recognized the existence of these new circumstances in a 2021 Communication of the European Commission by stating that: “A particularly cruel form of hybrid threat has emerged with the state-sponsored *instrumentalization* of people for political ends”. In the Strategic Compass for a Stronger EU Security and Defence by 2030, the EU recognised the instrumentalization of illegal migrations as a hybrid threat to the EU. ^2^ This recognition of changed circumstances drives a push for modifying state practice to protect state sovereignty at the expense of human rights and individual freedoms despite repeated warnings of those practices being contrary to international law. The European Court of Human Rights is currently adjudicating over 30 cases against EU Member States for pushback strategies against migrants in 2021 and 2023 ( Ginsburg, 2020). If enough liberal democratic states enshrine practices contrary to their international law commitments based on the arguments that facts and contexts have changed, therefore justifying their breaking of international commitments, it would provide Russia with more political arguments to back up its revisionist international law agenda.

Balancing the principles of the international protection of human rights with respect for state sovereignty over borders without either of those two being at the expense of the other is a demanding issue regarding instrumentalized migration cases. Target states must consider the full extent of the predicament in which instrumentalized migration leaves them. The phenomenon causes organizational and contingency challenges related to registering migrants, accommodating them, and transporting them in an orderly manner, often in contexts of limited resources. It can also create institutional stress linked to public opinion pressure in polarized societies, which are often disproportionately sensitive to a negative perception of the issue of migration. However, the deeper challenge that instrumentalized migration presents is a reflexive trap. As the examples above have shown, states adopt responses that end up undermining the principles of the protection of human rights and can cause democratic governance to weaken when adopting measures fundamentally contrary to international case law and commitments. This entrapment logic is important to the hybrid threat analysis since authoritarian states that instrumentalize migration flows against EU Member States seek to discredit European governance as a model.

## Avoiding the trap with the new migration pact?

The new EU Pact on Migration and Asylum is a policy framework that seeks to leverage the benefits presented by regular immigration pathways while simultaneously addressing the challenges associated with irregular migration. Migration crises in particular have triggered policy and legal initiatives regarding migration policies and their effectiveness, and especially the equilibrium between international law commitments and national sovereignty. The purpose of this section is to provide a selective perspective as to what the EU provides in terms of a toolbox for Member States facing instances of instrumentalized migration. Increasing and maintaining awareness of the tools available is critical to countering hybrid threats. Adequate responses to hybrid threat activities are ones in which the target state preserves a wide range of options for later use.

Especially since the Belarus crisis in 2021, instrumentalized migration began to be incorporated into EU legislative and non-legislative initiatives, indicating a greater political will to understand and manage the phenomenon. Notwithstanding the new Pact on Migration and Asylum, it is important to consider that the EU has a set of existing tools, such as: the toolbox addressing the use of commercial means of transport to facilitate irregular migration to the EU, the Union Civil Protection Mechanism, the EU Migration Preparedness and Crisis Management Network, and the Integrated Political Crisis Response arrangements. All those mechanisms contain provisions and funding that can also be mobilized in the event of any crisis per their relevant triggering procedures. Exploring the operational use of those mechanisms should provide more tools to Member States targeted by instrumentalized migration.

This part of the article discusses the European Commission’s Common Implementation Plan adopted on 12 June 2024, with ten interdependent building blocks and their potential implementation with a goal of understanding what can provide Member States with tools to prevent and absorb instrumentalized migration. EU Member States must submit their national plans for implementation to the Commission by December 2024. The Pact on Migration and Asylum includes specific measures to counter instrumentalized migration by providing EU Member States with a decision-making and activation procedure to trigger solidarity measures and support for crisis situations and *force majeure*. It contains a series of regulations updating and improving previous legislation in this area such as: the Asylum and Migration Management Regulation (AAMR), which provides for a better system in determining Member State responsibility for asylum applications and which contains the requirement for solidarity between Member States while faced with migratory pressure; the Asylum Procedure Regulation, including the Return Border Procedure Regulation, which streamlines asylum application decision procedures and establishes mandatory border procedures for asylum and return processes at external borders of the EU; and the Crisis and *Force Majeure* Regulation, which specifically deals with instrumentalization of migration and crisis situations, listing possibilities for derogations from international commitments and solidarity measures. The Pact also includes the Eurodac Regulation, the Screening Regulation, the Qualification Regulation, the Reception Conditions Directive, and the Union Resettlement Framework, which all constitute essential elements to a better streamlining of migration and asylum policies across the EU, thereby also reducing the attack surface for this particular hybrid threat activity. threat activity.

## Structural improvements and emergency and contingency tools

The Pact presents structural improvements to streamline migration and asylum policies and procedures. Block 1 is a substantial reconstruction of the European Asylum Dactyloscopy Database with more functionalities, a greater focus on international protection, increased categories for sea rescues, arrests, and more. Block 2 deals with rules for improved management of migration fluxes at the external borders of the EU. Among the various regulations are concerns regarding nonrefoulement and the imposition of stricter deadlines. Block 3 deals with reception conditions to balance appropriate treatment with the prevention of unauthorized movement. Block 4 aims to streamline decision making processes on asylum applications to ensure just, fair, and coherent asylum procedures. Block 9 contains a set of rules and regulations to enhance respect for fundamental rights and the right of asylum. Block 10 focuses on reinstallation, inclusion, and integration. Block 5 aims to incentivize returns and to make returns more effective. The role of Frontex would be of utmost importance while a manual to better implement and follow those procedures is being considered.

Blocks 7 and 8 contain the most relevant tools for countering instrumentalized migration as they provide for a solidarity mechanism that is flexible, but legally binding. Block 8 deals with contingency plans and crisis responses. Emergency plans need to be in place for solidarity to apply. At the core of Blocks 7 and 8 is the Crisis and *Force Majeure* Regulation. It provides for the possibility for a Member State to request the activation of a mandatory solidarity mechanism. In practical terms, a Member State that considers itself to be facing a situation of crisis or *force majeure* will be able to request an authorization from the European Commission to default from certain regulations in case of capacity overload. It will also be able to request solidarity from other Member States in addressing the situation. These cases concern occurrences of instrumentalized migration, but more generally, cases of disproportionate consequences outside the control of the Member State in question. The innovation in this regulation is that it provides a clear decision-making track: the requesting Member State must submit to the Commission a reasoned request establishing the grounds of the situation. The Commission will then assess the situation to determine its crisis character through an implementing decision process. It determines whether this is a situation of crisis including instrumentalization or *force majeure*. In particular, the Commission will assess why the Toolbox on Migration is not sufficient to address the situation. In consultation with the requesting Member State, the Commission includes a draft Solidarity Response Plan, tabling the needs in terms of relocation, financial solidarity, or alternative support. The Commission, in parallel with the implementing decision process mentioned above, makes a proposal to the Council authorizing derogations and establishing solidarity measures. This also contains a Solidarity Response Plan. At the same time, existing tools can be triggered by the Member State facing a situation of instrumentalized migration. The different levels of Integrated Political Crisis Response can be activated at the Council, paving the way for better information exchange and speeding up decisions on collective action. The effectiveness of those tools in avoiding the need for Member States to default from their international obligations will naturally depend on implementation and the usage being done of the decision-making procedure and solidarity process outlined above. States should seek to exhaust all possible solidarity measures before requesting authorizations to default from certain obligations contained in EU law or wider international commitments.

## Conclusions

The challenge posed by instrumentalized migration is strategic and countering it in the long-term demands an acute awareness of the precedence that EU Member States produce through their reaction and practice of democratic governance, as well as the current regime of international law. It is also equally important to build contingency measures to absorb the shocks created by instrumentalized migration, as this would have the potential to deny the effects sought after by the authoritarian states. Authoritarian states which instrumentalize migration pursue a goal of revising acceptable practices and the international law associated with them. When target states lack the resources to anticipate, prevent, and absorb the shocks caused by instrumentalized migration, they revert to policies and actions that fall short of commitments to the standard of international protection and international human rights law. This provides authoritarian states with a strong argument for pursuing their revisionist international law agenda of state supremacy over respect for human and individual rights. Authoritarian states can also claim that democratic states have double standards, and this would reduce their standing in international fora. The nature of the response to the challenge of instrumentalized migration is about preserving a margin of manoeuvre and a tool reservoir for the target states. The EU provides and frames a vast series of tools available for states to activate solidarity measures in order to collectively deny political effects of cases of instrumentalized migration against its Member States.

## Ethics and consent

Ethical approval and consent were not required.

## Data Availability

The data for this article consists of bibliographic references, which are included in the References section.

## Footnotes

^1^ According to UNHCR, “migrant” is widely used as an umbrella term to refer to a person who moves away whether internally or across border either voluntarily or by force. “Asylum-seeker” instead refers to person, who is seeking international protection. More information: Migrant definition | UNHCR; Asylum-seekers | UNHCR.

^2^ “State and non-state foreign actors are constantly refining and thereby strengthening their tactics, techniques and procedures, such as the *instrumentalization* of irregular migration, lawfare, as well as coercion targeting our economic and energy security.”
