# Supplementary material for: Instrumentalized migration: avoiding the trap
Source: Open Res Eur. 2025 Apr 29;4:251. Originally published 2024 Nov 22. [Version 2] doi: 10.12688/openreseurope.18635.2 (PMC12138498; doi:10.12688/openreseurope.18635.2)
Supplement: Peer review history

# Reviewer report: Reviewer response for version 2

**DOI:** 10.21956/openreseurope.21911.r54135

**Published:** 2025-05-30

**Authors:** Ayhan Kaya

**Affiliations:** 1 Istanbul Bilgi University, Istanbul, Turkey

The paper is very well written and introduces a new concept to the field: "instrumentalised migration." It also refers to the relevant literature and legal documents within the European framework. It dwells on the ways in which Belarus instrumentalised migration to put pressure on the neighbouring states and the EU in general, and how the New European Pact on Migration and Asylum offers some mechanisms for the member states to cope with such pressures, which might come from the authoritarian states outside the EU. I think the latest version of the paper is concise and updated, and convincing.

Is the argument persuasive and supported by appropriate evidence?

Partly

Is the work clearly and cogently presented?

Yes

Is the topic of the essay discussed accurately in the context of the current literature?

Yes

Does the essay contribute to the cultural, historical, social understanding of the field?

Yes

Reviewer Expertise:

Migration, Populism, Youth Radicalism, Islamophobism, Migrant Integration, Citizenship Studies, Nationalism Studies

I confirm that I have read this submission and believe that I have an appropriate level of expertise to confirm that it is of an acceptable scientific standard.

# Reviewer report: Reviewer response for version 2

**DOI:** 10.21956/openreseurope.21911.r53695

**Published:** 2025-05-29

**Authors:** Sofia Galani

**Affiliations:** 1 Panteion University of Social and Political Sciences, Athens, Greece

I am concerned that the authors have not sufficiently engaged with the reviewers' feedback. The main improvement is that the piece is now more appropriately referenced. Their revisions are not clearly reflected in the document. For instance, while Tunisia is not addressed at all, the mention of Morocco lacks a comparative analysis with Belarus and Russia. Additionally, the discussion of legal principles, though interesting, remains underdeveloped. Finally, the description of the EU framework is largely descriptive and lacks critical analysis.

Is the argument persuasive and supported by appropriate evidence?

Partly

Is the work clearly and cogently presented?

Partly

Is the topic of the essay discussed accurately in the context of the current literature?

Yes

Does the essay contribute to the cultural, historical, social understanding of the field?

Yes

Reviewer Expertise:

International law, human rights, law of the sea

I confirm that I have read this submission and believe that I have an appropriate level of expertise to confirm that it is of an acceptable scientific standard, however I have significant reservations, as outlined above.

# Reviewer report: Reviewer response for version 2

**DOI:** 10.21956/openreseurope.21911.r53696

**Published:** 2025-05-13

**Authors:** Federica Zardo

**Affiliations:** 1 Danube University, University for Continuing Education Krems, Krems, Austria

On the one hand, the authors say they have included the example of Tunisia, which is not correct because they only include the example of Morocco. Moreover, the case of Morocco in only mentioned i passing, but does not in any way provide a comparison. It is therefore not correct to state that there is a comparison between cases.

 The authors also state that they have included a critical assessment of the Pact, but the essay only describes it. At the moment, the only portion of text that "assesses the Pact" is the following: "The innovation in this regulation is that it provides a clear decision-making track: the requesting Member State must submit to the Commission a reasoned request establishing the grounds of the situation". 

 I would encourage the authors to explain how the different blocks of the Pact, or the crisis and force majeure Regulation if it is more appropriate, address the issues they identify.

 Moreover, what I still suggest is a clear statement or acknowledgment that the manuscript is an essay, to clarify that to the readers.

Is the argument persuasive and supported by appropriate evidence?

Partly

Is the work clearly and cogently presented?

Yes

Is the topic of the essay discussed accurately in the context of the current literature?

Yes

Does the essay contribute to the cultural, historical, social understanding of the field?

Yes

Reviewer Expertise:

NA

I confirm that I have read this submission and believe that I have an appropriate level of expertise to confirm that it is of an acceptable scientific standard, however I have significant reservations, as outlined above.

# Reviewer report: Reviewer response for version 1

**DOI:** 10.21956/openreseurope.20161.r47812

**Published:** 2025-02-18

**Authors:** Sofia Galani

**Affiliations:** 1 Panteion University of Social and Political Sciences, Athens, Greece

The article deals with an interesting and topical matter – that of instrumentalized migration. The authors explain in simple terms how instrumentalized migration is understood and draw on relevant examples, i.e. the practice of Belarus, to demonstrate how it works in practice. The authors also discuss the impact of instrumentalized migration on European democracies by relying upon what they call ‘the entrapment logic’ which as they explain force States to depart from their human rights obligations in the interest of protecting their State sovereignty. They then move on discussing how EU Member States can rely on the EU Pact on Migration and Asylum, which entered into force in June 2024, and especially the Crisis and Force Majeure Regulation in order to be better prepared for crises and avoid the “trap”.

 The article raises a lot of interesting and valid points. But, one may wonder whether it tries to do too much in too little space. The last two parts of the article simply list various tools that the EU Member States may use to deal with instrumentalized migration. This, however, does not leave much space to the authors to explain how these tools may enable the EU Member States to avoid the “trap” which seems to be at the heart of their argument. I’d therefore recommend the authors consider focusing more on the Crisis and Force Majeure Regulation, or at least be more specific and selective about which other aspect of the EU Pact on Migration and Asylum can help States deal with the crisis more effectively. 

 In addition, the authors seem to argue that authoritarian States instrumentalize migration in order to force democratic States to violate international law which challenges two principles of international law, Ex Injuria Non Oritur, and Ex Factis Jus Oritur. This is an interesting and innovative argument, but it is somehow lost in the current piece as it is “squeezed” between too many other points. Perhaps, the authors could consider leaving this point aside and working on another short piece elaborating more on this theoretical approach which merits further discussion. Instead, this piece could be used more to elaborate on the last two parts.

 With regards to the scope of the article, the authors may wish to shorten their introduction which is a bit repetitive and set out the aim of the paper more clearly. Perhaps, it would make sense to briefly mention the “entrapment logic” in the introduction as this comes in much later in the article and a reader may wonder what this “trap”, mentioned in the title of the article, really is. Readers may also benefit from a short explanation of, or at least a reference to, what a “hybrid threat” is.

 Another point that the authors may wish to reconsider is the reference to “forcibly displaced people” in the heading of the second part. The term “forcibly displaced” people is much more narrowly construed than the term “migrant” and several people affected by instrumentalized migration may, as a result, be left out of the scope of the discussion.

 Since the piece is focused on the EU, a reference to the case law of the CJEU, i.e. Case C‑72/22 PPU, 30 June 2022, would be more appropriate than – or equally necessary – to the reference to the European Court of Human Rights.

 Finally, the paper is thin in references. The authors could consider citing more relevant papers both in the interest of substantiating their claims but also for the benefit of the readers who may wish to study further relevant material. See, for example, Galani, Gkliati, Miholjcic Mitchell, or relevant EU and NATO reports.[Ref 1, 2 and 3]

Is the argument persuasive and supported by appropriate evidence?

Partly

Is the work clearly and cogently presented?

Partly

Is the topic of the essay discussed accurately in the context of the current literature?

Yes

Does the essay contribute to the cultural, historical, social understanding of the field?

Yes

Reviewer Expertise:

International law, human rights, law of the sea

I confirm that I have read this submission and believe that I have an appropriate level of expertise to confirm that it is of an acceptable scientific standard, however I have significant reservations, as outlined above.

## References

[1] : The Instrumentalization of Migration as a Hybrid Threat against the EU: Can Human Rights Become ‘Hybrid’ Too?.2024; 10.1163/9789004707993_011 263-286 10.1163/9789004707993_011

[2] : Let’s Call It What It Is: Hybrid Threats and Instrumentalisation as the Evolution of Securitisation in Migration Management. *Insight Instrumentalisation of Migrants, SanctionsTackling Hybrid Attacks and Schengen Reform in the Shadows of the Pact* . Reference source

[3] : Migration as an Instrument of Modern Political Warfare: Cases of Turkey, Morocco and Belarus. *Jean Monnet Network on EU Law Enforcement Working Paper Series* . Reference source

# Reviewer report: Reviewer response for version 1

**DOI:** 10.21956/openreseurope.20161.r47279

**Published:** 2024-12-28

**Authors:** Natalia Letki, Paweł Kaczmarczyk

**Affiliations:** 1 Centre of Migration Research, University of Warsaw, Warsaw, Poland; 2 University of Warsaw, Warszawa, Poland

This is an interesting and well written, but brief opinion piece and not a research article. It makes two main arguments: that by limiting human rights, e.g. the right to asylum, liberal democracies deny their core values and become complicit in the actions of authoritarian states, and that solidarity measures in the new Pact on migration and asylum could be used to prevent EU Member States from giving in to the spiral of instrumentalization of migration. While both arguments make a lot od sense, they seem fairly obvious and therefore they do not constitute an original contribution. The paper does not provide any evidence to prove usefulness of Pact's solidarity measures to prevent instrumentalization; while relocational and fiscal solidarity are indeed present in the Pact, we do not know whether, and if yes - when and how - they will be implemented. The Authors also do not address the fact that some MSs oppose the Pact and openly use instrumentalization to their own political benefits despite the availability of solidarity measures. 

 There is more evidence needed for some of the claims the Authors make. For example, what is the source of information on the national composition of irregular migrants on the PL/BY border? Also, the Finnish legal acts cited have not — to our knowledge — been implemented. 

 While the discussion of *Ex Injuria Non Oritur *and *Ex Factis Jus Oritur *principles is beyond our competence to evaluate, we feel this is where the most interesting potential contribution of the paper might be - to demonstrate, in reference to legal and political discourse and practice of the MSs, how these principles and the tension between them are being deployed. However, this would require the discussion of the Pact to be much more detailed and less cursory.

Is the argument persuasive and supported by appropriate evidence?

Partly

Is the work clearly and cogently presented?

Yes

Is the topic of the essay discussed accurately in the context of the current literature?

Partly

Does the essay contribute to the cultural, historical, social understanding of the field?

Partly

Reviewer Expertise:

Public opinion, policy preferences, migration studies

We confirm that we have read this submission and believe that we have an appropriate level of expertise to state that we do not consider it to be of an acceptable scientific standard, for reasons outlined above.

# Reviewer report: Reviewer response for version 1

**DOI:** 10.21956/openreseurope.20161.r47817

**Published:** 2024-12-26

**Authors:** Federica Zardo

**Affiliations:** 1 Danube University, University for Continuing Education Krems, Krems, Austria

The article is a well-informed and thoughtful essay examining how the EU Pact on Migration and Asylum seeks to address the growing phenomenon of instrumentalized migration. It begins with the premise that the instrumentalization of migration by non-EU states is on the rise, with the primary aim of shifting the balance between adherence to international law and the protection of state sovereignty. The authors contend that recent cases, such as those involving Belarus (2021) and Russia (2023), illustrate coercive tactics employed by authoritarian regimes to exploit migration flows as tools for manipulating the political and legal responses of target states.

 The essay highlights a significant challenge faced by European states: a "reflexive trap," wherein their responses to such hybrid threats risk undermining international commitments. This, in turn, provides authoritarian actors with a platform to claim success, eroding the credibility of democratic governance. To counter this, the authors propose that the EU Pact on Migration and Asylum offers promising structural reforms and emergency mechanisms. These include solidarity measures and the Crisis and Force Majeure Regulation, which collectively aim to bolster resilience and mitigate the impact of such hybrid threats.

 The essay is well-written and draws on key literature about coercive engineered migration and instrumentalization. However, its use of supporting sources could be further enriched by incorporating the latest analyses of the Belarus case, that also critically reflect on the EU´s reaction to instrumentalization cases, such as:

- Grześkowiak, M. (2023). The “Guardian of the Treaties” is No More? The European Commission and the 2021 Humanitarian Crisis on Poland–Belarus Border. Refugee Survey Quarterly, 42(1), 81–102. Ref 1
- Gkliati, M. (2023). Let’s Call It what It Is: Hybrid Threats and Instrumentalisation as the Evolution of Securitisation in Migration Management. European Papers - A Journal on Law and Integration, 2023 8(2), 561–578. Ref 2 
- Kelemen, & Pavone. (n.d.). Where Have the Guardians Gone? Law Enforcement and the Politics of Supranational Forbearance in the European Union by R. Daniel Kelemen, Tommaso Pavone: SSRN. Retrieved 10 August 2023, from Ref 3 

The cases analyzed in the essay are undoubtedly relevant. The authors describe them as "prototypes of instrumentalized migration," but I would suggest referring to them as "exemplary cases." Furthermore, I would encourage the authors to reflect on and briefly discuss how these cases compare to other instances of instrumentalized migration, such as those involving Turkey, Morocco, or Tunisia. Can the underlying hypotheses or rationales driving the actions of these different “instrumentalizing actors” also apply to other cases? If so, how? If not, why not? Such reflections could provide a broader comparative framework and enrich the analysis.

 While the essay does include some citations, and as an essay it naturally allows for personal interpretations and opinions, certain statements and assumptions would benefit from stronger referencing. This is the case, for instance, of the following sentences:

- The short-term consequences of instrumentalized migration may be severe, but the end goal of the adversary is to interlink those implications with longer term consequences.
- Migrants were prohibited from ever returning to Russia at the cost of threats on the wellbeing of their families and of forced conscription or other disproportionate measures in retaliation to any future irregular stay on Russian territory.
- In this sense, the protection of state sovereignty at the expense of individual rights and liberties is at the core of the authoritarian agenda”
- The new EU Pact on Migration and Asylum is a policy frame work that seeks to leverage the benefits presented by regular immigration pathways while simultaneously addressing the challenges associated with irregular migration.

The tension between the two legal principles, *Ex Injuria Non Oritur* and *Ex Factis Jus Oritur* forms the core of the article's argument. I would suggest that this pivotal theme be introduced earlier, ideally in the introduction, to frame and drive the entire analysis. Highlighting this tension upfront would provide a clearer focus for the essay and help readers understand how the legal and policy debates are situated within this broader framework. In the analytical section, I recommend providing more detailed explanations of how the EU Pact on Migration and Asylum specifically addresses the tension between these two principles. Additionally, the section should expand on how the provisions of the Pact reduce the EU’s vulnerability to instrumentalizing actors. For instance, it would be valuable to explain how the structural and emergency mechanisms, such as solidarity measures and the Crisis and Force Majeure Regulation, directly mitigate the strategic goals of those employing migration as a tool of coercion.

Is the argument persuasive and supported by appropriate evidence?

Partly

Is the work clearly and cogently presented?

Yes

Is the topic of the essay discussed accurately in the context of the current literature?

Yes

Does the essay contribute to the cultural, historical, social understanding of the field?

Yes

Reviewer Expertise:

Migration Studies, International Relations.

I confirm that I have read this submission and believe that I have an appropriate level of expertise to confirm that it is of an acceptable scientific standard, however I have significant reservations, as outlined above.

## References

[1] : The “Guardian of the Treaties” is No More? The European Commission and the 2021 Humanitarian Crisis on Poland–Belarus Border. *Refugee Survey Quarterly* .2023;42(1) : 10.1093/rsq/hdac025 81-102 10.1093/rsq/hdac025

[2] : Testing a signal-processing image analysis technology in hospital on suspicious pigmented naevi referred for biopsy. *J Vis Commun Med* .2024; 10.1080/17453054.2024.2438635 1-6 10.1080/17453054.2024.2438635 39720991

[3] : Where Have the Guardians Gone? Law Enforcement and the Politics of Supranational Forbearance in the European Union. *SSRN Electronic Journal* .2021; 10.2139/ssrn.3994918 10.2139/ssrn.3994918

# Reviewer report: Reviewer response for version 1

**DOI:** 10.21956/openreseurope.20161.r47816

**Published:** 2024-12-19

**Authors:** Luisa Marin, Michele Gigli

**Affiliations:** 1 University of Insubria, Varese, Italy; 2 European University Institute, Fiesole FI, Italy

The article discusses how instrumentalized migration threatens the EU and its Member States’ system of migration management, and tries to explain how this threat can be overcome. The purpose of the article is two fold: first, understanding the challenge posed by instrumentalized migration as a ‘hybrid threat’ activity; then, presenting the tools provided by the EU to avoid the trap of instrumentalized migration.

 a)         The practice of instrumentalized migration has been presented in an interesting and innovative manner. The authors described how authoritarian states use instrumentalized migration to force democratic governments to choose whether to abide to their obligations of international protection at the sake of political credibility or to safeguard state sovereignty disregarding their commitment to the rule of law. The authors use the recent Russian and Belarusian crises as prototypes of instrumentalized migration, and find that the entrapment logic of instrumentalized migration arises from a new tension between two principles on international law which did not exist when those principles were adopted. The analysis is well rooted in the relevant literature and make an important contribution to the legal understanding of this topic.

 b)         The analysis of the tools provided by the EU to avoid the trap of instrumentalized migration lacks necessary specificness to make a compelling argument. First of all, the article refers to the Pact on migration and asylum, presented in 2020, while in 2024 the European legislator has adopted legislative instruments expression of that Pact. This part [Structural improvements and emergency and contingency tools] requires a more in-depth analysis of the relevant instruments approved in 2024 by the European legislator. Currently the article touches upon the policy choices or policy goals chosen by the legislator, yet it does not look closely at the manner these choices and goals have been translated into legal provisions.

 In particular, the following questions remain unanswered: why discussing only the migration pact and no other tools? (Is this innovative? Has it been adopted on purpose? Is it the newest/ most suitable one? Are there other tools at all?); how does/ can the EU specifically contrast instrumentalized migration?

 The authors’ argument seems to be that most efficient solidarity measures at EU level help avoiding the effects of politicized migration against Member States, yet these solidarity mechanisms are only presented in their stated aims and not examined nor discussed in their functioning. Nevertheless, the need for solidarity is a long-standing issue in the discussion on how to improve migration management in the EU (mandatory solidarity in migration management has also been invoked on the basis of art. 80 TFEU). Therefore, this part seems rather to answer the more general question “how to better manage migration?”, while the specificness of instrumentalized migration remains unaddressed. Moreover, it is true that the new migration pact includes the ‘Crisis and Force Majeure Regulation’ and the solidarity mechanism, but these tools have been criticized in literature for not being compelling enough to ensure effective solidarity. This article does not contrast these critics. This part of the analysis seems to be necessary against the background of the stated aims of the article. If there were more political cohesion among Member states, instrumentalized migration would presumably be less impactful, but experts agree that such a level of political cohesion is just not yet there.

 In conclusion, while the conceptual analysis of instrumentalized migration is interesting and fairly problematized, what is missing is a convincing claim on how to respond to instrumental use of migration policies for political purposes: the analysis of the European instruments remains somehow limited to the stated policy goals. We recommend the authors to be more forceful on the elements that contribute to the concluding argument, namely that a higher degree of political cohesion among Member States would neutralize the effects of instrumentalized migration.

Is the argument persuasive and supported by appropriate evidence?

Partly

Is the work clearly and cogently presented?

Partly

Is the topic of the essay discussed accurately in the context of the current literature?

Yes

Does the essay contribute to the cultural, historical, social understanding of the field?

Yes

Reviewer Expertise:

NA

We confirm that we have read this submission and believe that we have an appropriate level of expertise to confirm that it is of an acceptable scientific standard, however we have significant reservations, as outlined above.
